# m6A‐Modified circTET2 Interacting with HNRNPC Regulates Fatty Acid Oxidation to Promote the Proliferation of Chronic Lymphocytic Leukemia

**DOI:** 10.1002/advs.202304895

**Published:** 2023-10-11

**Authors:** Zijuan Wu, Xiaoling Zuo, Wei Zhang, Yongle Li, Renfu Gui, Jiayan Leng, Haorui Shen, Bihui Pan, Lei Fan, Jianyong Li, Hui Jin

**Affiliations:** ^1^ Department of Hematology the First Affiliated Hospital of Nanjing Medical University Jiangsu Province Hospital Nanjing Medical University Nanjing 210029 China; ^2^ Key Laboratory of Hematology of Nanjing Medical University Nanjing 210029 China; ^3^ Jiangsu Key Lab of Cancer Biomarkers, Prevention and Treatment Collaborative Innovation Center for Personalized Cancer Medicine Nanjing Medical University Nanjing 210029 China; ^4^ Anqing First People's Hospital of Anhui Medical University Anqing First People's Hospital of Anhui Province Anqing 246004 China; ^5^ Department of Hematology Affiliated People's Hospital of Jiangsu University Zhenjiang 212002 China; ^6^ National Clinical Research Center for Hematologic Diseases the First Affiliated Hospital of Soochow University Suzhou 215000 China

**Keywords:** chronic lymphocytic leukemia, circTET2, circular RNA, fatty acid oxidation, HNRNPC, N6‐methyladenosine (m6A), proliferation

## Abstract

Chronic lymphocytic leukemia (CLL) is a hematological malignancy with high metabolic heterogeneity. N6‐methyladenosine (m6A) modification plays an important role in metabolism through regulating circular RNAs (circRNAs). However, the underlying mechanism is not yet fully understood in CLL. Herein, an m6A scoring system and an m6A‐related circRNA prognostic signature are established, and circTET2 as a potential prognostic biomarker for CLL is identified. The level of m6A modification is found to affect the transport of circTET2 out of the nucleus. By interacting with the RNA‐binding protein (RBP) heterogeneous nuclear ribonucleoprotein C (HNRNPC), circTET2 regulates the stability of CPT1A and participates in the lipid metabolism and proliferation of CLL cells through mTORC1 signaling pathway. The mTOR inhibitor dactolisib and FAO inhibitor perhexiline exert a synergistic effect on CLL cells. In addition, the biogenesis of circTET2 can be affected by the splicing process and the RBPs RBMX and YTHDC1. CP028, a splicing inhibitor, modulates the expression of circTET2 and shows pronounced inhibitory effects. In summary, circTET2 plays an important role in the modulation of lipid metabolism and cell proliferation in CLL. This study demonstrates the clinical value of circTET2 as a prognostic indicator as well as provides novel insights in targeting treatment for CLL.

## Graphical abstract

M6A‐modified circTET2 modulated by RBMX and YTHDC1 binds to HNRNPC and promotes its translocation from nucleus to cytoplasm. CP028 modulates the expression of circTET2 and promotes the apoptosis of CLL cells. The RNA‐protein complex in cytoplasm activates the mTORC1 signaling pathway and interacts with the 3′ end of CPT1A mRNA to regulate the FAO of CLL cells, which co‐contributes to the development of CLL.

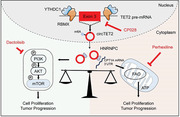

## 1 Introduction

Tumor growth is a dynamic process and metabolic reprogramming is a hallmark of tumor cells.^[^ ^1^ ^]^ Chronic lymphocytic leukemia (CLL), a well‐known malignant hematological disease with great clinical heterogeneity, is also characterized by metabolic heterogeneity.^[^ ^2^ ^]^ The adaptation of CLL cells' metabolism is more likely to be dependent upon lipid metabolism, which is one of the most prominent metabolic alterations in cancer because of the abundant lipid deposits when compared with normal B cells.^[^ ^3^ ^]^ Although accumulating evidence reveals that altered lipid metabolism is associated with CLL disease progression and treatment responsiveness,^[^ ^4^ ^]^ the extent of its heterogeneity and relationship to molecular heterogeneity has not been systematically studied.

Circular RNAs (circRNAs) are single‐stranded and covalently closed RNA molecules that are categorized as non‐coding RNAs (ncRNAs) and are ubiquitously distributed across species, ranging from viruses to mammals.^[^ ^5^ ^]^ Most research has demonstrated that circRNAs are involved in the fate of tumors in various ways, such as by acting as miRNA sponges and protein decoys and by encoding peptides.^[^ ^6^ ^][^ ^7^ ^]^ Previously, we identified the significance of circ_0132266, circ‐RPL15, and mitochondrial genome‐derived circRNA in CLL.^[^ ^8^ ^]^ However, the current understanding of the regulatory mechanism of circRNAs in CLL is limited to ceRNA, and whether it could interact with RNA‐binding proteins is still unclear. Increasing evidence shows that circRNAs regulate the lipid metabolism of tumor cells and participate in the development of disease, providing an innovative basis for novel clinical biomarkers and targeted therapeutic strategies.^[^ ^9^ ^]^ For example, circACC1 improved the stability and activity of AMPK in colorectal cancer, promoting glycolysis and fatty acid oxidation (FAO) to maintain energy balance;^[^ ^10^ ^]^ in laryngeal squamous cell carcinoma, the low expression of hsa_circ_00 33988 is related to fatty acid degradation.^[^ ^11^ ^]^ CircH19 has been noted to regulate the nucleocytoplasmic transport and transcription functions of SREBP1c protein by binding to PTBP1, further affecting the transcription and protein expression of downstream adipogenic genes, thereby regulating adipocyte differentiation and lipid metabolism.^[^ ^12^ ^]^ Although our previous study revealed that circ‐RIC8B regulated the lipid metabolism through the miR199b‐5p/LPL axis,^[^ ^13^ ^]^ the function and mechanism of circRNAs are still not very clear in CLL. In addition, N6‐methyladenosine (m6A) modification is the most abundant epi‐transcriptomic modification of circRNAs and plays an important role in maintaining the biological activity of circRNAs.^[^ ^14^ ^]^ The study of m6A modification in circRNAs has thus recently become a significant area of research and needs to be explored and fully understood in CLL.

In the present study, we constructed an m6A scoring system and demonstrated its significance in the prognosis of CLL patients with our own data and an external independent dataset. Furthermore, an m6A‐related circRNA prognostic signature was established for the first time by analyzing the whole transcriptional sequencing results of 53 CLL patients, and we found m6A‐modified circTET2 to be an indicator of the prognosis of patients with CLL. RNA‐binding proteins (RBPs) potently and ubiquitously regulate transcripts by modulating the process of RNA synthesis, alternative splicing, modification, transport, and translation.^[^ ^15^ ^]^ We herein demonstrated that the biogenesis and modulation of circTET2 were affected by the RBPs RBMX and YTHDC1, and screened‐out splicing inhibitor CP028 could regulate their expressions. Besides, circTET2 interacting with HNRNPC occupied a vital role in the modulation of lipid metabolism and the progression of CLL. Last but not least, we found that inhibitors of mTOR and FAO exert a synergistic effect on CLL cells. We expect these findings to provide novel insights into the targeted treatment of CLL.

## 2 Results

### 2.1 The Significance of m6A and Construction of a Risk Model According to m6A‐Related Circrnas in CLL Patients

**Figure 1:**
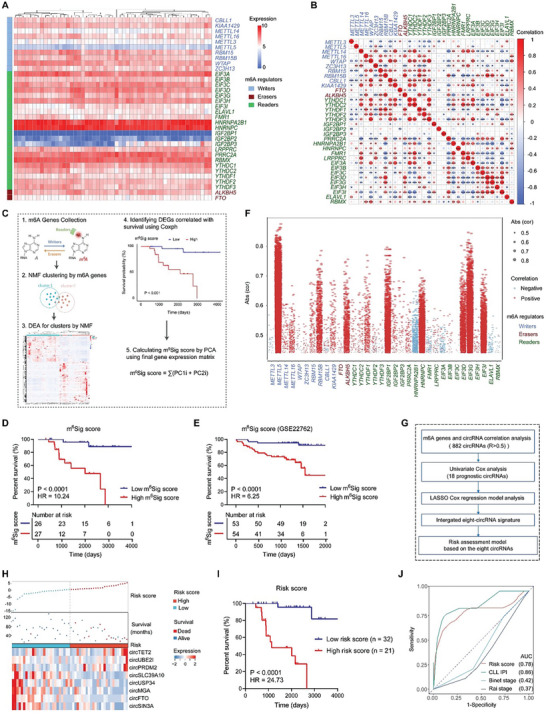
m6A modification in CLL and a risk model presented for m6A‐related circRNAs. A) The expression patterns of m6A regulators in CLL patients (*n =* 53). B) Correlations among the m6A genes. *p < 0.05, **p < 0.01, ***p < 0.001. C). Workflow of the m^6^Sig score construction. D) Kaplan‐Meier curves for patients with high and low m^6^Sig scores (*n =* 53). E. Survival analysis of m^6^Sig score in the collected independent CLL cohort (GSE22762, *n =* 107). F) m6A regulators and related circRNAs. G)Workflow for the construction of our risk model of m6A‐related circRNAs. H) Upper panel, distribution of samples in the high‐ and low‐risk score groups; middle panel, OS of each sample; lower panel, the expression pattern of eight prognostic signatures in the two groups. I) Kaplan–Meier curve of the OS of patients in the high‐ and low‐risk score groups. J) ROC curve analysis of the risk score model (*n =* 53).

To determine the significance of m6A modification in CLL, we conducted whole‐transcriptome sequencing of a cohort of 53 newly diagnosed CLL patients, and 34 m6A regulators were included in the analysis. The expression patterns of m6A regulators and their expression correspondence were exhibited separately in **Figure** **1**A,B. Next, through a detailed workflow, we performed consensus clustering with non‐negative matrix factorization (NMF) to identify distinct m6A modification patterns based on the expression of 34 m6A regulators. The result indicated that m6A regulators were correlated with the heterogeneity and prognosis of CLL. Therefore, we constructed an m6A scoring system and calculated the m6A signature score (m^6^Sig score) Figure 1C, Figure S1A,B, Supporting Information. The m^6^Sig score displayed potential predictive value for prognosis (area under the curve (AUC) = 0.85 at 5 years, Figure S1C, Supporting Information, and patients with a low m^6^Sig score had a prominent survival benefit Figure 1D. The specificity and sensitivity of the m^6^Sig score were validated by integrating the clinical characteristics and genomic information from TCGA CLL database GSE22762, Figure S1D, Supporting Information. Consistently, patients with a higher m^6^Sig score had shorter overall survival (OS) Figure 1E. We also explored the differential status of these m6A regulators in CLL.^[^ ^16^ ^]^ Analysis of genetic datasets encompassing 537 CLL patients showed an occurrence of gene abnormalities in m6A regulators Figure S1E, Supporting Information. To further construct a risk model according to m6A‐related circRNAs, we screened circRNAs with a correlation coefficient greater than 0.5 Figure 1F,G. CLL patients were categorized into low‐ and high‐risk groups according to risk score. Eight m6A‐related circRNAs (circTET2, circUBE2I, circPRDM2, circMGA, circUSP34, circSIN3A, circSLC39A10, and circFTO) were ultimately selected to constitute the risk model Figure 1H, and we noted that patients in the high‐risk group had shorter OS relative to the low‐risk group Figure 1I. The risk score also showed a higher AUC in CLL patients Figure 1J.

### 2.2 Upregulated Expression of m6A‐Modified circTET2 is Related to the Prognosis of CLL Patients

**Figure 2:**
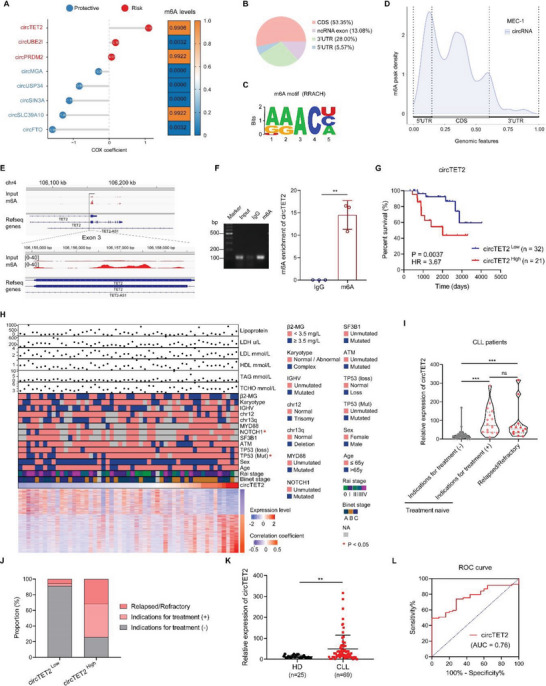
m6A‐modified circTET2 is up‐regulated in CLL and related to the prognosis of patients. A) Left, individual risk score of the eight circRNAs in the risk model. Right, m6A levels of the eight circRNAs as predicted by circBank. B) The distribution of m6A peaks. C) Predominant consensus motifs identified with m6A‐seq peaks. D) Density of m6A methylation peaks in circRNAs. E. m6A peak via meRIP‐seq on TET2 exon 3 as visualized by IGV. F) Methylated RNA in cells was immunoprecipitated with an m6A antibody, followed by qPCR analyses with primers against circTET2. Error bars represent the means±SD derived from three independent experiments. Statistical analyses were performed using a two‐tailed Student's t‐test, **p < 0.01. G) Kaplan–Meier curves for high and low circTET2 levels (*n =* 53). H) Clinical characteristics of the enrolled CLL patients (*n =* 53); the lower heatmap comprised genes that correlated with circTET2. I)The expression levels of circTET2 in CLL patients with different statuses. No indication for treatment (*n =* 40), Indication of treatment (*n =* 16), Relapsed/Refractory (*n =* 13). J) Proportion of patients with different statuses in high and low circTET2 expression groups. K) The expression levels of circTET2 in CLL patients (*n =* 25) and CD19+ B cells from healthy volunteers (*n =* 69). L) ROC curve analysis showed the diagnostic value of circTET2 in CLL.

The risk score of circTET2 ranked first in the model. The CircBank database revealed that circTET2, circPRDM2, and circSLC39A10 exhibited higher m6A levels **Figure** **2**A, and SRAMP predicted that the eight circRNAs would possess m6A modification sites Figure S2A, Supporting Information. MeRIP‐seq was exploited to explore m6A modification in the CLL cell line MEC‐1. A proportion of m6A peak distributions displayed m6A peaks in the coding sequence (CDS), 3′ untranslated region (UTR), 5′ UTR, and ncRNA exon Figure 2B. AAAC was detected as the predominant consensus motif in MEC‐1 cells Figure 2C and m6A peaks were abundant in the CDS, especially near the start codons Figure 2D. The m6A peaks for circTET2 and circPRDM2 were more abundant in the m6A group than in the IP group (Figure 2E, Figure S2B, Supporting Information). Thus, circTET2 with the highest risk score as well as predicted m6A level was then selected in our study. RNA immunoprecipitation following qPCR (RIP‐qPCR) with a divergent primer for circTET2 was implemented to confirm the m6A modification (Figure 2F). Survival analysis revealed that patients with higher expression of circTET2 had a much shorter OS (Figure 2G). We summarized participant characteristics and observed that the expression of circTET2 significantly correlated with the appearance of NOTCH1 and TP53 mutations (Figure 2H). Clinical samples from 69 CLL patients, including treatment‐naïve and relapsed/refractory (R/R) patients, were then collected to detect the expression of circTET2. Among these samples, the levels of circTET2 were the lowest in samples from patients who showed no indications for treatment (Figure 2I). The expression level of circTET2 was then ranked and divided into two groups. CLL patients with higher circTET2 expression were more likely to be treated. In addition, patients in the circTET2 high group had a higher proportion of experiencing relapsed/refractory stage (Figure 2J). Twenty‐five healthy donors were subsequently collected to determine the expression of circTET2. Compared with normal CD19+ B cells, circTET2 was dramatically upregulated in CLL patients (Figure 2K). The expression of circTET2 was also assessed in multiple cancer cell lines from diffuse large B cell lymphoma (DLBCL), cutaneous T‐cell lymphoma (CTCL), and acute myeloid leukemia (AML) in addition to CLL, and our results showed that the levels of circTET2 in CLL were significantly higher than in other groups (Figure S3, Supporting Information). In view of the upregulated circTET2 expression in CLL, we generated a receiver‐operating characteristic (ROC) curve to evaluate its potential diagnostic value in effectively distinguishing CLL patients from healthy individuals. The AUC of circTET2 reached 0.76 (Figure 2L), indicating that circTET2 might be a potential biomarker in the screening of CLL.

### 2.3 Characteristics of circTET2 in CLL

**Figure 3:**
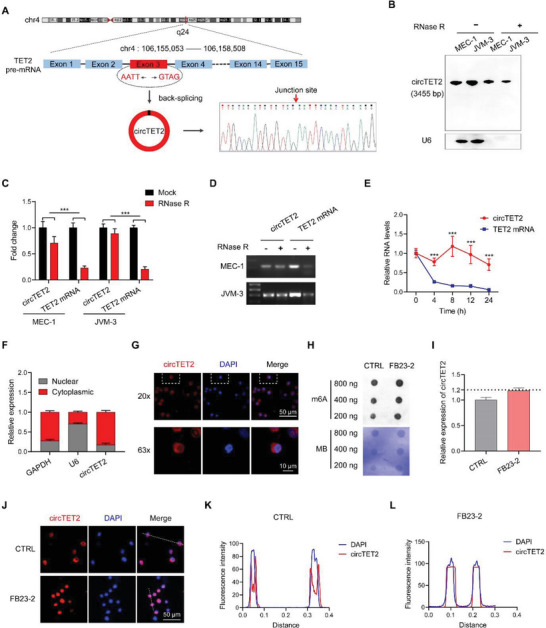
Characterization of circTET2 in CLL. A) The genomic loci of TET2 and the “head‐to‐tail” splicing of circTET2 from exon 3 verified by Sanger sequencing following PCR. B. Northern blot using a junction‐specific DIG‐labeled probe shows the endogenous existence of circTET2 in MEC‐1 and JVM‐3 cells. The expression levels of the linear and circular form of TET2 with the treatment of RNase R as detected by qRT‐PCR (C) and agarose gel electrophoretic assays (D). E) The abundances of circTET2 and linear TET2 with actinomycin D treatment in MEC‐1 cells. F) Nucleocytoplasmic separation assays detected the distribution of circTET2 in MEC‐1 cells. G) FISH assay shows the location of circTET2 in MEC‐1 cells. Scale bar, Upper: 50 µm, Lower: 10 µm. H) Determination of m6A abundance in MEC‐1 cells upon FB23‐2 treatment for 72 h via dot blot assay. Methylene Blue (MB) represents the loading control of RNA samples. I) The change in circTET2 levels in MEC‐1 cells treated with FB23‐2. J) IF staining images of circTET2 in MEC‐1 cells treated with or without FB23‐2. Scale bar, 50 µm. K, L) Colocalization analysis of circTET2 and DAPI with Image (J) software. Error bars represent the means±SD derived from three independent experiments. ***p < 0.001.

A divergent primer was designed to amplify circTET2 located on chr4q24, and Sanger sequencing validated the head‐to‐tail splicing of exon 3 (**Figure** **3**A). Use of northern blotting (Figure 3B), RNase R treatment (Figure 3C,D), and actinomycin D assay (Figure 3E, Figure S4A, Supporting Information) demonstrated the circular form of circTET2 and the stability of circTET2. Nucleocytoplasmic separation (Figure 3F, Figure S4B, Supporting Information H) and FISH (Figure 3G, Figure S4C, Supporting Information) assay revealed that circTET2 was principally located in the cellular cytoplasm. To explore whether the expression and location of circTET2 were affected by the m6A modification, we treated cells with a demethylase inhibitor, FB23‐2. Upon FB23‐2 treatment, the m6A level in cells was elevated while the circTET2 expression showed no significant change (Figure 3H,I). However, circTET2 was abundantly distributed in the nucleus (Figure 3J–L). These findings suggested that the transport of circTET2 to the cytoplasm was somewhat dependent on m6A modification.

### 2.4 RBMX and YTHDC1 Regulate the Biogenesis of circTET2

**Figure 4:**
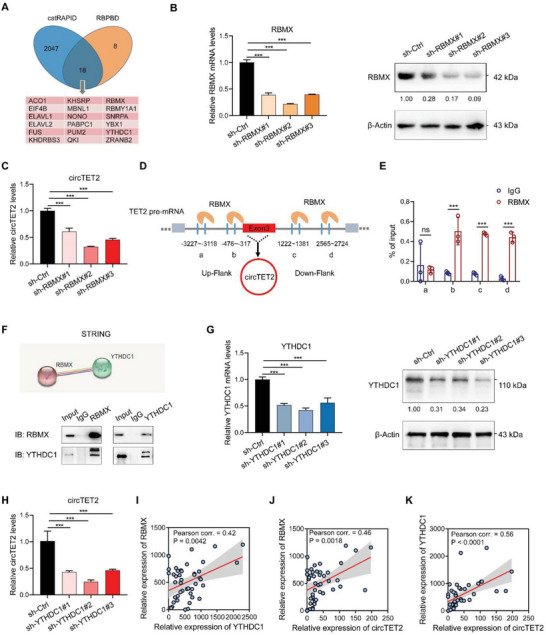
RBMX and YTHDC1 regulate the biogenesis of circTET2. A) Predicted RBPs bind to the flanking introns of circTET2 predicted by catRAPID (http://s.tartaglialab.com/page/catrapid_group) and RBPBD (http://rbpdb.ccbr.utoronto.ca/). B) Knockdown efficiency of RBMX detected by qRT‐PCR and western blot assays. C) The change in circTET2 levels. D,E) Binding sites for RBMX on the flanking introns predicted by catRAPID (http://s.tartaglialab.com/page/catrapid_group) (D) and validated by RIP‐qPCR (E). F) Co‐IP was adopted to detect the protein–protein interactions between RBMX and YTHDC1. G) Knockdown efficiency of YTHDC1 as determined with qRT‐PCR and western blot assays. H) The change in circTET2 levels. I) Correlations between RBMX and YTHDC1 expression in CLL patients as analyzed by Pearson analysis (*n =* 44). J) Correlations between circTET2 and RBMX expression (*n =* 44). K)Correlations between circTET2 and YTHDC1 expression (*n =* 44). Data represent the mean ± SD. ns, not significant, *p < 0.05, **p < 0.01, ***p < 0.001.

CircRNA is regarded as an unusual product of alternative splicing, and RBPs can bind to flanking introns of circRNAs, thus serving as splicing factors.^[^ ^17^ ^]^ To investigate the potential RBPs involved in this process, the database catRAPID and RBPBD were then applied (**Figure** **4**A). Among the 18 predicted RBPs, we discerned that splicing factors RBMX and YTHDC1 bound to the flanking sequences of circTET2, and this attracted our attention. shRNAs were then designed to knock down RBMX, and this reduction significantly impaired the expression of circTET2 (Figure 4B, C). Binding sites of RBMX (a, b, c, d) on the flanking introns were obtained by catRAPID (Figure 4D), and RIP‐qPCR analysis displayed the binding of RBMX and TET2 on b, c, and d sites (Figure 4E). YTHDC1 is an interacting partner of RBMX and was reported to affect the nuclear export of methylated mRNAs and circRNAs.^[^ ^18^ ^]^ Co‐immunoprecipitation (co‐IP) assay verified the interaction between RBMX and YTHDC1 (Figure 4F), and the silencing of YTHDC1 weakened the expression of circTET2 as expected (Figure 4G,H). However, alteration in the location of circTET2 was not observed after knocking down YTHDC1 (Figure S5, Supporting Information). When we evaluated the levels of RBMX and YTHDC1 in CLL patients, we observed a positive correlation between YTHDC1 and RBMX or circTET2, as well as between circTET2 and RBMX (Figure 4I,K).

### 2.5 CircTET2, Which Promotes Cellular Proliferation, is Involved in the Regulation of the mTORC1‐Signaling Pathway and Lipid Metabolism

**Figure 5:**
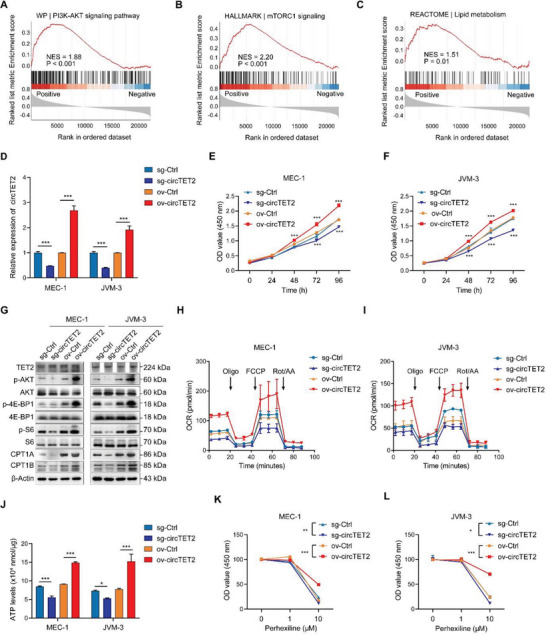
CircTET2 promotes cell proliferation and is involved in regulating the mTORC1‐signaling pathway and FAO. A–C)Gene set enrichment analysis (GSEA) shows the signaling pathways enriched in genes that are positively related to circTET2. NES, normalized enrichment score. D) Knockdown and overexpression efficiency of circTET2. E,F)CCK8 assay shows the proliferative viability of cells with different circTET2 levels. G) Protein levels determined in cells with reduced or increased circTET2 levels. H,I) Oxygen consumption rate (OCR) as detected by seahorse assays. J) ATP levels of cells with different expression levels of circTET2. K,L) CCK8 shows the viability of cells treated with perhexiline for different time periods and for circTET2 overexpressing and knockdown cells treated with perhexiline for 24 h. Data represent the mean ± SD from three independent experiments. *p < 0.05, **p < 0.01, ***p < 0.001.

To explore a role for circTET2 in CLL, we conducted gene set enrichment analysis (GESA) of circTET2‐associated genes, and our results revealed that circTET2 was involved in the PI3K‐AKT pathway (**Figure** **5**A) and mTORC1 signaling (Figure 5B), as well as in lipid metabolism (Figure 5C). We then constructed stable CLL cell lines with circTET2 overexpression or silencing (Figure 5D). When we first conducted CCK8 analysis, results illustrated that circTET2 promoted the proliferation of CLL cells (Figure 5E,F). From these results, we observed that the change in circTET2 did not alter TET2 protein levels, whereas overexpression of circTET2 activated the mTORC1 pathway and enhanced the levels of CPT1A and CPT1B, while knockdown of circTET2 showed opposite results (Figure 5G). Seahorse assay revealed that overexpression of circTET2 in CLL cells led to a higher oxygen consumption rate (OCR), representing FAO levels (Figure 5H,I). ATP levels were also determined and the consistent results demonstrated that circTET2 promoted FAO (Figure 5J). The FAO inhibitor (FAOi) perhexiline maleate was subsequently used to validate the role of mTORC1 signaling and FAO in the proliferation of CLL cells. Perhexiline inhibited cell viability in a dose‐dependent manner, and the inhibitory effect was dampened by circTET2 overexpression (Figure 5K,L). These data suggested that FAO was increased and indispensable for sustaining CLL cellular proliferation.

### 2.6 CircTET2 Regulates CLL Cells in a HNRNPC‐CPT1A‐Dependent Manner

**Figure 6:**
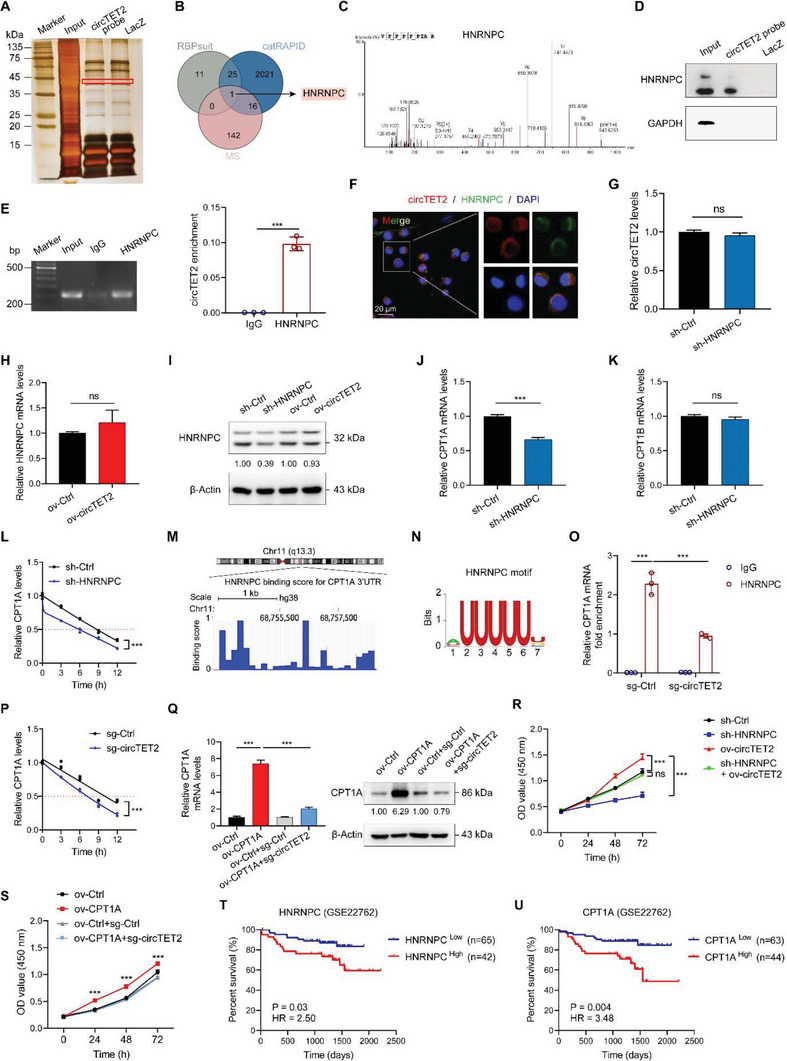
The circTET2 and HNRNPC complex interact and stabilize CPT1A mRNA. A) Silver stain shows the proteins pulled down by the circTET2 probe. B) The Venn diagram shows the potential RBPs that bind to circTET2. C) Peak map of HNRNPC acquired from the RNA pulldown mass spectrometry assay. D) Protein pulled down by circTET2 probe with HNRNPC antibody was detected by western blotting. E) RIP assay shows the interaction between HNRNPC and circTET2. F) FISH‐IF assay shows the co‐localization of circTET2 and HNRNPC (scale bar, 20 µm). G) The change in circTET2 levels after HNRNPC knockdown. H) HNRNPC mRNA levels after circTET2 overexpression. I) HNRNPC protein levels after HNRNPC knockdown. J,K) The change in CPT1A and CPT1B mRNA levels after HNRNPC knockdown. L) Degradation rates of CPTA1 mRNA in cells with HNRNPC knockdown. M) HNRNPC binding sites in the CPT1A 3′UTR region as predicted by RBPsuit. N) Motif of HNRNPC. O) RIP assay shows the interaction between HNRNPC and CPT1A. P)Degradation rates of CPTA1 mRNA in cells with circTET2 knockdown. Q) The relative expression of CPT1A after overexpression of CPT1A and knockdown of circTET2 as detected by qRT‐PCR and western blot assays. R) Growth curves of cells with circTET2 overexpression and/or HNRNPC knockdown. S) Proliferative ability of cells with CPT1A overexpression and circTET2 knockdown. T,U) Survival analysis of HNRNPC (T) and CPT1A (U) in collected independent CLL cohort (GSE22762, *n =* 107). Error bars represent the means±SD derived from three independent experiments. ns, not significant, ***p < 0.001.

Accumulating evidence depicts circRNAs as exerting functions via their interaction with RNA‐binding proteins (RBPs).^[^ ^19^ ^]^ To explore the RBPs that bind with circTET2, we implemented mass spectrometry following RNA pull‐down (**Figure** **6**A, Table S1, Supporting Information). Bioinformatic analysis using RBPsuit and catRAPID was also conducted to screen the possible binding proteins for circTET2, and we determined that HNRNPC was the most likely RBP (Figure 6B,C). We then confirmed the interaction between circTET2 and HNRNPC by RNA pull‐down (Figure 6D). RIP assay demonstrated the enrichment of circTET2 in the complex precipitated with antibody against HNRNPC (Figure 6E). FISH‐immunofluorescence (FISH‐IF) analysis also verified the co‐localization of the two molecules (Figure 6F). When physically bound to each other, we noted that circTET2 and HNRNPC exerted no influence on their mutual expression (Figure 6G–I). As CPT1A and CPT1B may be modulated by circTET2, we then assessed their changes after depletion of HNRNPC and noted that the expression of CPT1A but not of CPT1B was downregulated after knocking down HNRNPC (Figure 6J,K). Cytoplasmic HNRNPC was reported to consistently regulate the stability of target genes. We then performed actinomycin D assay and confirmed that HNRNPC modulated the stability of CPT1A (Figure 6L). Starbase showed that HNRNPC contained potential binding sites in the 3′UTR of CPT1A. The application of RBPmap further validated this result (Figure 6M), and through RBPsuit we acquired the HNRNPC motif (Figure 6N). RIP assay was then conducted and confirmed the predicted results (Figure 6O). More importantly, the interaction between HNRNPC and CPT1A mRNA was significantly reduced after knocking down circTET2 (Figure 6O). Further results demonstrated that circTET2 had an effect on the stability of CPT1A (Figure 6P). We then overexpressed CPT1A and knocked down circTET2, and the elevated CPT1A level was observed to be suppressed (Figure 6Q). To confirm whether circTET2 functioned through HNRNPC and CPT1A, we performed a CCK8 assay and found that circTET2 was crucial for the reduced cellular proliferative capability induced by sh‐HNRNPC (Figure 6R). Additionally, CCK8 assay also confirmed that cellular viability induced by CPT1A was significantly depleted with the silencing of circTET2 (Figure 6S). Subsequently, the OS based on the expression of HNRNPC and CPT1A was conducted. Predictably, higher HNRNPC and CPT1A levels predicted poorer OS for patients with CLL (Figure 6T,U). Collectively, these results indicated that circTET2 regulated CLL cells in a HNRNPC‐CPT1A‐dependent manner.

### 2.7 Effects of CP028, Dactolisib, and Perhexiline on CLL cells

**Figure 7:**
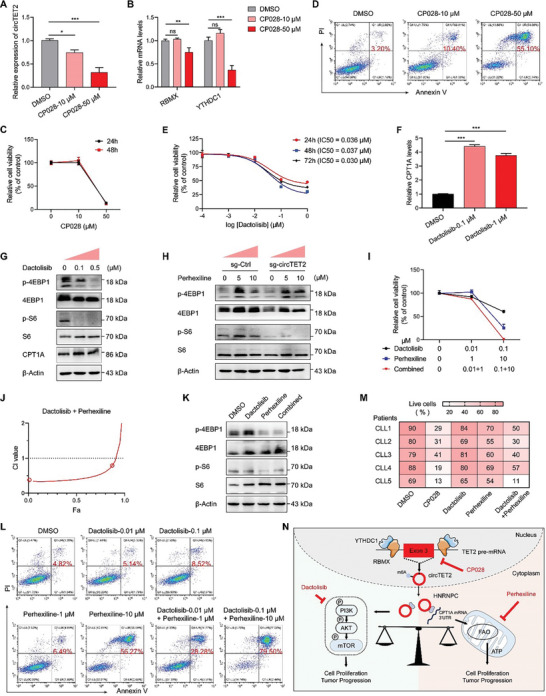
Effects of CP028, dactolisib, and perhexiline on CLL cells. A) The change in circTET2 levels with CP028 treatment for 24 h. B) Relative expression of RBMX and YTHDC1 in MEC‐1 cells treated with CP028. C) CCK8 was used to detect the viabilities of cells treated with CP028. D) Apoptotic rate of cells treated with CP028 for 24 h. E) IC50 of dactolisib in MEC‐1 cells treated for different time periods. F)qRT‐PCR analysis shows the expression change in CPT1A after 24 h of treatment with dactolisib. G) The protein levels for CPT1A and the mTOR pathway in cells with dactolisib treatment. H) The expression of mTOR pathway proteins with circTET2 knockdown and perhexiline treatment. I,J) The inhibitory effects of dactolisib and perhexiline and the combination index were calculated by CompuSyn. K) Apoptotic rate of cells treated with CP028, dactolisib, and/or perhexiline for 24 h detected with flow cytometry. L) The protein levels of the mTOR pathway in cells treated with dactolisib and or perhexiline. M) Trypan blue staining was used to evaluate the apoptotic rate of primary cells from five CLL patients. N) Schematic representation of circTETE2 promoting cell proliferation by modulating FAO and mTOR signaling. Error bars represent the means±SD derived from three independent experiments. ns, not significant, *p < 0.05, **p < 0.01, ***p < 0.001.

Given the oncogenic role of circTET2 in CLL, targeting circTET2 was hypothesized to constitute a potential therapeutic strategy. Targeting splicing factors is regarded as a novel therapeutic strategy for tumors,^[^ ^20^ ^]^ and since circTET2 was modulated by the splicing process and RBPs, we attempted to uncover splicing factor inhibitors that would target circTET2. We thereby applied five inhibitors: indisulam (targeting splicing by inducing RBM39 degradation); H3B‐8800 (a modulator of the SF3b complex); and the three pre‐mRNA splicing inhibitors isoginkgetin, madrasin, and CP028. Of these, CP028 significantly reduced the levels of circTET2 (**Figure** **7**A, Figure S6A, Supporting Information). In addition, the expression of RBMX and YTHDC1 was determined in cells after treatment with the splicing inhibitors, and we demonstrated that CP028 attenuated RBMX and YTHDC1 expression while the others did not (Figure 7B, Figure S6B, Supporting Information). The consistent results we observed with circTET2 indicated that RBMX and YTHDC1 were potential regulators of the splicing process and the circularization of circTET2. The significant inhibition of cellular viability and promotion of cell apoptosis were noted with the use of CP028 (Figure 7C,D), while the other four inhibitors also showed pronounced inhibition (Figure S6C, Supporting Information). The dual ATP‐competitive PI3K and mTOR inhibitor dactolisib were also used to dramatic effect on MEC‐1 cells (Figure 7E). However, although the application of dactolisib induced the expression of CPT1A, it downregulated the levels of p‐4EBP1 and p‐S6 (Figure 7F, G). Treatment with perhexiline activated the phosphorylation of p‐4EBP1 and p‐S6, which was alleviated by circTET2 silencing (Figure 7H). Thus, we discerned a synergistic role for dactolisib and perhexiline and uncovered their significantly enhanced effect (Figure 7I,J). The augmented levels of p‐4EBP1 and p‐S6 were also downregulated with the combinatorial use of dactolisib (Figure 7K). Flow cytometric analysis confirmed the marked apoptotic rate with the drug combination compared with single‐drug treatment (Figure 7L). The synergistic drug actions we noted were further validated with primary cells from CLL patients using trypan blue staining (Figure 7M), and these results indicated their potential therapeutic strategy in CLL.

## 3 Discussion

In recent years, the research progress of epigenetics, including DNA/RNA modification, histone modification, and chromatin rearrangement, has greatly enriched the understanding of physiological and pathological processes.^[^ ^21^ ^]^ Among them, m6A is the most common post‐transcriptional modification and has been confirmed to participate in the biosynthesis and regulation of coding RNA or non‐coding RNA, affecting the occurrence, development, and outcome of diseases.^[^ ^22^ ^]^ However, how m6A modification plays a regulatory role in CLL has not been systematically studied. Herein, we reveal the significance of m6A in the prognosis of CLL based on the m^6^Sig score, which indicates that m6A modification may be involved in patients’ disease progression and therapeutic outcomes.

m6A regulators have been widely reported to be involved in the regulation of leukemia. For example, the m6A demethylase METTL3 promotes the occurrence of AML and serves as a potential therapeutic target.^[^ ^23^ ^]^ Fat mass and obesity‐associated protein (FTO) may act as an oncogene to promote leukemia and inhibit all‐trans retinoic acid‐mediated leukemia cell differentiation.^[^ ^24^ ^]^ Targeting m6A modification was verified to be significant in potentially refining clinical therapy.^[^ ^25^ ^]^ In this study, we established an m^6^Sig score system and confirmed that patients with a lower m^6^Sig score were characterized by prolonged survival time. Through screening the m6A‐related circRNAs based on whole‐transcriptome sequencing results of 53 CLL patients, we constructed a risk prognosis assessment model and revealed its clinical value as a prognostic molecular marker for CLL patients. Within the model, circTET2 was highly expressed in CLL patients, and a strong association with prognosis was identified. There are limits to referring circTET2 as a biomarker for CLL, as genetic characteristics vary in patients from different centers and regions.^[^ ^26^ ^]^ Our previous study has documented the significant differences in IGHVDJ gene usage, mutations, and stereotypy between Chinese and Italian patients.^[^ ^27^ ^]^ Thus, efforts are still needed for the clinical application of circTET2, including enlarging the cohort numbers not only in our own centers but also in centers from other regions.

CircRNAs are described as special products of RNA alternative splicing, meaning that the biogenesis of circRNAs depends on the classical splicing mechanism.^[^ ^28^ ^]^ Numerous RBPs that belong to the family of splicing factors modulate the circular process of circRNAs. Accumulating evidence has demonstrated that the aberrant splicing events that promote disease progression provide potential therapeutic targets. Multiple inhibitors of splicing factors show excellent efficacy on CLL cells. Here, we found that the back‐splicing of exon 3 was affected by the inhibitor splicing factor CP028, resulting in the modulation of the expression of circTET2. RBMX and YTHDC1 are described not only as RBPs but also as m6A regulators and are influenced by CP028 as well. They are also widely noted to play pivotal roles in the splicing of mRNAs.^[^ ^29^ ^]^ Recently, Chen, et al. revealed that YTHDC1 facilitated circNSUN2 export from the nucleus to the cytoplasm.^[^ ^30^ ^]^ However, in the present study, we observed the binding of RBMX and YTHDC1 on the flanking sequences of circTET2 and confirmed that the two jointly promoted the splicing and circulization of exon 3, which forms circTET2. The location determines the molecular functions of circRNA and recent studies show that m6A modification occurs in circRNA to control its nuclear transport.^[^ ^30^, ^31^ ^]^ However, the underlying mechanism is not clearly understood. In this research, we found that increased m6A levels restricted the transport of circTET2 from the nucleus to the cytoplasm. Cytoplasmic circTET2 was the one involved in the regulation of CLL cells. Upon treatment with a demethylase inhibitor, circTET2 tended to be impeded in the nucleus, which restricted the function of circTET2 and inhibited cell proliferation. We proposed that the spatial structure of circRNA may contribute to this process. According to our previous study, circRNAs are not a single‐strand continuous loop, and they probably have a double‐strand structure, which may be dynamically reversible and have an impact on their mechanism and functions.^[^ ^32^ ^]^ m6A modification in circRNAs could make them compressed or loose in space, which thus affects their export from the nucleus into the cytoplasm.

CLL is a hematological malignancy characterized by clonal proliferation of mature B lymphocytes.^[^ ^33^ ^]^ To meet the energy demands during the proliferative process, CLL cells could drive metabolic reprogramming.^[^ ^34^ ^]^ Genes involved in metabolic pathways were found to be up‐regulated in CLL compared with normal lymphocytes.^[^ ^35^ ^]^ However, recurrent genetic mutations in CLL cells have not been shown to be directly involved in altering metabolic pathways, suggesting that metabolic reprogramming may not be directly induced by genetic mutations.^[^ ^36^ ^]^ In this study, we discovered that circTET2 interacting with HNRNPC activated the PI3K‐AKT‐mTORC1 signaling pathway and accelerated the process of FAO via CPT1A.

Activation of Akt was noted to occur more frequently in aggressive CLL.^[^ ^37^ ^]^ We have previously reported the upregulation of mROTC1‐related proteins in CLL patients, especially those with refractory or relapsed disease.^[^ ^38^ ^]^ With the treatment of the dual ATP‐competitive PI3K and mTOR inhibitor dactolisib, the proliferative ability of CLL cells was observed to be significantly impaired. In addition, the expression of CPT1A was also reduced. It is known that ATP mainly comes from oxidative phosphorylation, glycolysis, or FAO. CircTET2 induces the production of ATP through FAO to provide the metabolic needs in the process of CLL cell proliferation. This indicates that CLL cells may be more dependent on FAO to satisfy the energy supply. Inhibition of FAO was confirmed to significantly reduce the drug resistance of CLL cells and improve the clinical outcomes of patients. Perhexiline, a FAO inhibitor, shows safety in long‐term treatment compared with etomoxir.^[^ ^39^ ^]^ The viability of CLL cells was indeed weakened after treatment with perhexiline. As expected, the killing effect of dactolisib was strengthened when combined with perhexiline. These results are in line with the evidence that cellular metabolic reprogramming in CLL tends to utilize lipids, and FAO is vital in aggressive CLL.^[^ ^40^ ^]^ To summarize, in this study we first documented the clinical significance of the m^6^Sig score in CLL. We then discovered that m6A‐modified circTET2 modulated by RBMX and YTHDC1 binded to HNRNPC and promoteed its translocation from the nucleus to the cytoplasm. CP028 modulated the expression of circTET2 and promoted the apoptosis of CLL cells. The RNA‐protein complex in the cytoplasm activated the mTORC1 signaling pathway and interacted with the 3′ UTR of CPT1A mRNA to regulate the FAO of CLL cells, which co‐contributed to the development of CLL (Figure 7N).

## 4 Conclusion

In summary, we demonstrated the prognostic significance of m6A in CLL patients and identified m6A‐modified circTET2 as a prognostic marker in CLL. CircTET2 which was upregulated in CLL was modulated by the splicing factors RBMX and YTHDC1. Interacting with HNRNPC, circTET2 appeared to be involved in the regulation of FAO and the mTORC1 signaling pathway to provide energy demands and promote the proliferation of CLL cells. The combined inhibition of mTOR and FAO showed an enhanced effect, which suggested a novel therapeutic strategy for the treatment of CLL. The data generated by this study collectively provide new evidence for a role and underlying mechanism for circRNAs in CLL lipid metabolism, and this may engender novel potential targets in clinical treatment.

## 5 Experimental Section

### m^6^Sig Score Calculation

m6A‐related genes, including 10 writers, 2 erasers, and 22 readers, were collected from the literature. Using the CancerSubtypes Bioconductor package, CLL samples were divided into two categories through non‐negative matrix factorization (NMF) based on the expression of m6A‐related genes to identify distinct m6A modification patterns. Differential expression analysis of the two categories was performed using edgeR and differentially expressed genes (DEGs) were defined as those with fold change ≥ 1.5 and *p*‐value < 0.05. Next, univariate Coxph regression analysis was conducted on these DEGs with the survival package based on CLL OS time. The genes with a potential prognostic impact (*p* < 0.05) were selected further through recursive feature elimination (RFE) with a random forest model and the “boot” method in the caret package. Principal component analysis (PCA) was conducted on the expression profiles of final gene sets, and principal components 1 and 2 were summed as the signature score. If the signature score passed the evaluation, the final gene sets were defined as the m6A signature and its score was presented as the m^6^Sig score.

### m^6^Sig score Evaluation and Validation

To evaluate the association between the m^6^Sig score and CLL prognostic outcome, ROC analysis was performed using the TimeROC package, and the respective AUCs were compared with the random performance (AUC = 0.5). To identify the m^6^Sig score or the clinical indices of CLL with an independent prognostic effect, a univariate and multivariate Cox proportional hazard model (Coxph) was applied using the survival package. The Kaplan–Meier distribution was computed using the survminer package, and the comparison of survival distributions grouped by the m^6^Sig score median value was performed using the log‐rank method.

### Independent Datasets for the m^6^Sig score

To validate the m6A signature obtained from its own CLL cohort, the GEO database was searched and found a dataset, GSE22762, containing gene expression profiles with OS data. To reduce the batch effects, only the largest cohort (*n =* 107) from three microarray platforms were selected. Then, ROC analysis, univariate Coxph, and KM analysis were performed.

### Establishment of Prognostic Model of circRNAs Associated with m6A

The correlation coefficient (R) between circRNAs and the m6A gene was calculated according to the normalized expression. If *R* > 0.5, the circRNA was considered to be related to m6A. Univariate Cox regression analysis determined its prognostic value for OS, and *p* < 0.05 was considered significant. Lasso penalty Cox regression analysis was used for further screening, and eight circRNAs were finally selected. Multivariate Cox analysis was used to establish a prognosis model. All patients were divided into high‐ and low‐risk groups, with the median risk score as the critical value. The contributions of the eight circRNAs in the risk model were also analyzed.

### Clinical Samples, Cell Lines, and Reagents

Peripheral blood mononuclear cells (PBMCs) from CLL patients were collected and whole‐transcriptome sequencing was performed as previously reported.^[^ ^38^ ^]^ The CLL cell lines MEC‐1 and JVM‐3, human B lymphocyte cell lines GM12878 and NCI‐BL2009, DLBCL cell lines SU‐DHL‐10 and WSU‐DLCL2, T cell lymphoma cell lines H9 and MT4, CML cell line K562, and monocytic leukemia cell line THP1 were used in this study and cultured in RPMI‐1640 with 10% FBS (BioChannel Biological Technology) and 1% PS (Gibco), and cell transfection was performed as previously reported.^[^ ^41^ ^]^ We purchased short hairpin RNAs and overexpression vectors from Genechem (Shanghai, China). For circTET2 knockdown, the CRISPR‐Cas 13 method was adopted, and mixed three sgRNAs to target circTET2. The sequence of shRNAs or sgRNAs is listed in Table S2, Supporting Information. Small molecular inhibitors and other reagents are listed in Table S3, Supporting Information.

### Cell Transfection

The lentiviral vectors for stable knockdown of RBMX, YTHDC1, HNRNPC, circTET2, and circTET2 overexpression were purchased from Geneseed Biotech (Guangzhou). Among them, the CRISPR/Cas13 (Cas13) system was used for circTET2 knockdown. Cas13 was a class of RNA‐mediated targeted RNA cutting systems that had been widely used in the fields of RNA knockdown, RNA single base editing, RNA site‐specific modification, RNA live cell tracing, and nucleic acid detection. Compared with traditional RNA interference techniques, the Cas13 system offered distinct advantages over knockdown efficiency and specificity. All these lentivirus infections were performed according to the manufacturer's protocol. Briefly, 200 000 cells were seeded in a 6‐well plate, and an infection‐enhancing agent, Hitrans G, was added. Lentivirus vectors were then added to cells with a MOI of 100. After 16 h, cells were collected and cultured with a fresh medium. To increase the transfection efficiency, infected cells were further treated with puromycin (1 µg mL^−1^) for several days.

### Northern Blot Analysis

RNA (10–20 mg) used for the detection of endogenous circTET2 was denatured and loaded on 1% agarose gel, and electrophoresis was conducted at 25 V overnight at 4 °C. RNA was transferred on a Hybond N+ membrane (Amersham, USA) by capillarity action for 20 h after being washed in 20 × SSC. Prehybridization and hybridization were performed in 10.0 ml of DIG Easy Hyb with denatured probes designed by Saicheng Biotechnology (Guangzhou, China) at 68 °C (for 2 h and overnight, respectively). The sequence of the circTET2 probe was as follows: GCCAUCCACAAGGCUGCCCUCUAGUUGAAUUCUACACAUCUGCAAGAUGGGAAAUCAUAUUGAGUCUUGACAGGUGUA. The membrane was then washed and blocked and finally exposed on phosphorimager screens for analysis.

### RNA Preparation and qRT–PCR

For RNase R treatment, 2.5 µg of total RNA was incubated with RNase R (3 U/µg) (Epicentre Technologies, Madison, WI, USA) for 20 min at 37 °C. For actinomycin D treatment, the culture medium was added with actinomycin D (2 ug mL^−1^), and cells were collected at a specified time to assess the stability of circRNA. The nuclear and cytoplasmic fractions were extracted using a PARIS Kit (Life Technologies, USA). Total RNAs were extracted with TRIzol (Ambion, USA) according to the manufacturer's instructions and reverse‐transcribed using a HiScript III 1st Strand cDNA Synthesis Kit (Vazyme, Shanghai, China). The quantification of RNAs was determined using a ChamQ SYBR qPCR Master Mix (Vazyme), and primers are listed in Table S2, Supporting Information.

### Western Blotting

The total protein that was extracted using RIPA lysis buffer was applied for SDS‐PAGE and transferred to a PVDF membrane (Millipore). The membrane was incubated with the primary antibody at 4 °C overnight and the subsequent secondary antibody at room temperature for 1 h. Primary antibodies against AKT (#9272), p‐AKT (#4060), 4E‐BP1(#9644), p‐4E‐BP1 (#2855), p70S6K (#2708), p‐p70S6K (Thr389, #9234), and RBMX (#14794) were purchased from Cell Signaling Technology (Danvers, MA, USA). TET2 (#21207‐1‐AP), CPT1A (#15184‐1‐AP), CPT1B (#22170‐1‐AP), and HNRNPC (#11760‐1‐AP) antibodies were purchased from Proteintech (China). Antibodies against YTHDC1 (#ab264375) and β‐actin were from Abcam (Cambridge, UK) and Beyotime (Shanghai, China), respectively.

### Fluorescence in situ Hybridization (FISH) and Immunofluorescence

Cells were prepared and fixed in 4% paraformaldehyde and permeabilized with Triton X‐100. The primary antibody to HNRNPC (Proteintech, 11760‐1‐AP, China) was added after discarding the blocking reagent, and the secondary antibody was added the next day. After 1 h of incubation, cells were pre‐hybridized and then mixed with a hybridization solution that contained Cy3‐labeled circTET2 probes, while cell nuclei were stained with 4,6‐diamidino‐2‐phenylindole (DAPI, Beyotime, China). The images were photographed under confocal laser‐scanning microscopy (Zeiss, LSM5 Live, Germany).

### m6A Dot Blot

RNA samples with or without FB23‐2 (HY‐127103, MedchemExpress) treatment were denatured at 95 °C for 3 min and spotted onto Amersham Hybond‐N+ membranes (GE Healthcare), followed by UV cross‐linking. After blocking in phosphate‐buffered saline (PBS) with Tween 20 containing 5% bovine serum albumin for 1 h, the membrane was incubated with an anti‐m6A antibody (Synaptic Systems) at 4 °C overnight. The next day, the membrane was incubated with HRP‐conjugated anti‐mouse immunoglobulin G (Abcam) for 1 h at room temperature. Finally, the membrane was visualized with a Chemiluminescent HRP substrate kit (Millipore, USA). The other membrane stained with 0.02% methylene blue (Solarbio, China) was used to indicate the total input RNA content.

### Seahorse Assay

Oxygen consumption rate (OCR) was measured using a Seahorse XFe96 Analyzer (Seahorse Bioscience, Agilent Technologies, Santa Clara, CA, USA) as previously reported.^[^ ^38^ ^]^ The experiment was performed with three biological replicates, and data were expressed as the mean ± SD. Two‐way ANOVAs and t‐tests were used to perform statistical analysis.

### ATP Detection

ATP was determined with an ATP Assay Kit (Beyotime, #S0026, Shanghai, China) according to kit guidelines. Cells were collected and lysed with lysis buffer and then centrifuged at 12 000 g for 5 min. The supernatant was transferred and mixed with ATP detection buffer. The ATP concentration was calculated using the luminescence value according to the standard curve. The experiment was performed with three biological replicates, and data were expressed as the mean ± SD. Two‐way ANOVAs and t‐tests were used to perform statistical analysis.

### Chromatin Isolation by RNA Purification (ChIRP)‐MS

The ChIRP‐MS was conducted exactly as described previously.^[^ ^42^ ^]^ The biotin‐labeled probe was pre‐treated and incubated with the supernatant extracted from the cells, and Dynabeads MyOne Streptavidin C1 beads (Thermo Scientific) were then added. The RNA–protein mixture was washed and boiled in SDS buffer followed by mass spectrometry (BIOTREE, Shanghai) or western blot detection.

### Cell Viability and Apoptosis Assays

To evaluate cellular viability, cells treated with inhibitors were seeded in 96‐well plates and incubated with CCK‐8 (APExBIO, Houston, TX, USA) for 3 h. For apoptotic assays, cells treated with inhibitors were stained with Annexin V‐FITC and PI (Yeasen, #40 302, Shanghai, China). The ratio of apoptotic cells was then evaluated by flow cytometry (Cytoflex, Beckman Coulter, Brea, CA, USA).

### Co‐Immunoprecipitation (Co‐IP)

Cells were incubated with NP‐40 lysis buffer (Beyotime, #P0013F, Shanghai, China) for 30 min followed by washing twice with PBS. After centrifugation at 14, 000 g for 5 min, the supernatant was collected and 100 µL was used as input. The other aliquots were incubated with antibodies and rotated overnight at 4°C. Thirty microliters of Protein A + G agarose (Beyotime, #P2012) were added the next day and rotated for 2 h. The supernatant was discarded by centrifugation and then washed the mixture with NP‐40 containing PMSF and cocktail. After washing three times, the mixture was resuspended with loading buffer and heated up to 100 °C for 5 min. The samples were then used in the immunoblot assay.

### RNA Immunoprecipitation (RIP)

RIP experiments were performed using the Magna RIP RNA‐Binding Protein Immunoprecipitation Kit (Millipore, Bedford, MA, USA) following the manufacturer's instructions. Twenty million cells were collected and lysed for each reaction. Magnetic beads were prepared and incubated with antibodies against HNRNPC (Proteintech, China), YTHDC1 (Abcam, #ab264375, Cambridge, UK), RBMX (Cell Signaling Technology, #14 794, Danvers, MA, USA), or IgG with rotation for 30 min at room temperature. Beads were then washed and resuspended with RIP IP buffer and rotated together with RIP lysate overnight. Each immunoprecipitated fraction with proteinase K buffer was resuspended and the tube was shaken at 55 °C to digest the protein. Supernatants were transferred to a new tube and RNA purification was executed. The abundance of circTET2 was ultimately detected by qRT‐PCR assay.

### Statistical Analysis

Statistical analysis was performed with GraphPad Prism 8. Data were shown as mean ± SD of three independent biological replicates. Statistical analyses were performed as described in the figure. Differences were considered significant based on p‐values (*p < 0.05, **p < 0.01, ***p < 0.001).

### Ethics Approval Statement and Patient Consent Statement

Peripheral blood samples were obtained from CLL patients and healthy volunteers at the First Affiliated Hospital of Nanjing Medical University, Jiangsu Province Hospital (Nanjing, China). The experiments were undertaken with the understanding and written consent of each subject. The use of human samples was approved by the institutional ethics committee (2023‐SR‐172).

## Conflict of Interest

The authors declare no conflict of interest.

## Supporting information

Supporting Information

## Data Availability

The data that support the findings of this study are available from the corresponding author upon reasonable request.
