# Supplementary material for: m6A‐Modified circTET2 Interacting with HNRNPC Regulates Fatty Acid Oxidation to Promote the Proliferation of Chronic Lymphocytic Leukemia
Source: Adv Sci (Weinh). 2023 Oct 11;10(34):2304895. doi: 10.1002/advs.202304895 (PMC10700176; doi:10.1002/advs.202304895)
Supplement: Supplementary file 1 — Supporting Information [file ADVS-10-2304895-s001.pdf]

ADVANCED SCIENCE
Open Access

Supporting Information

for *Adv. Sci.*, DOI 10.1002/advs.202304895

m6A-Modified circTET2 Interacting with HNRNPC Regulates Fatty Acid Oxidation to Promote the Proliferation of Chronic Lymphocytic Leukemia

Zijuan Wu, Xiaoling Zuo, Wei Zhang, Yongle Li, Renfu Gui, Jiayan Leng, Haorui Shen, Bihui Pan, Lei Fan, Jianyong Li* and Hui Jin**

Supporting Information

m6A-modified circTET2 interacting with HNRNPC regulates fatty acid oxidation to promote the proliferation of chronic lymphocytic leukemia

Zijuan Wu[1,2,3], Xiaoling Zuo[3,4], Wei Zhang[1,2,3], Yongle Li[1,2,3], Renfu Gui[1,2,3], Jiayan Leng[5], Haorui Shen[1,2,3], Bihui Pan[1,2,3], Lei Fan[1,2,3], Jianyong Li[1,2,3,6*], Hui Jin[1,2,3*]*

Supplementary Figures

Figure S1

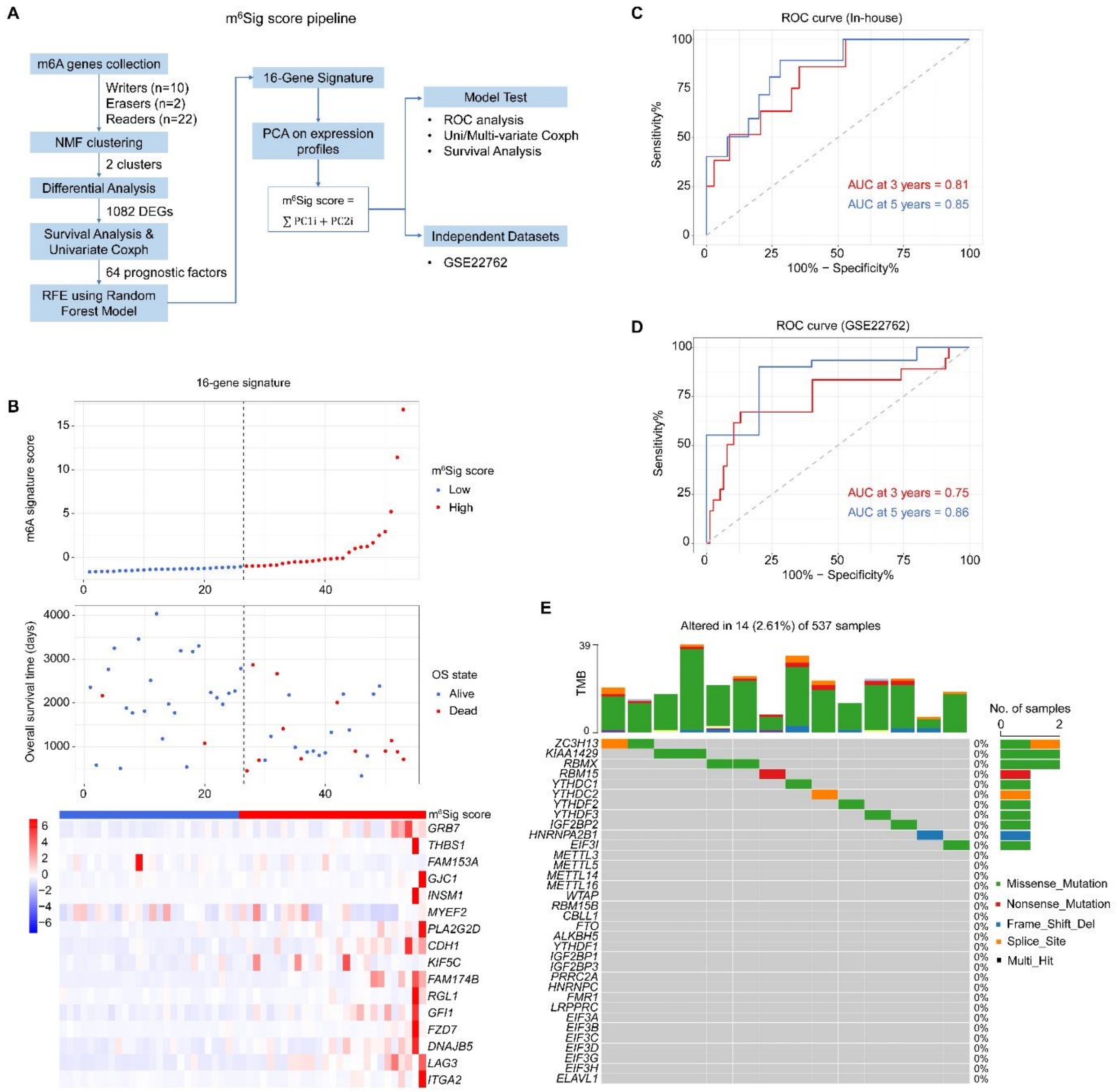

Figure S1. Significance of m6A modification in CLL. A. The calculation and evaluation of m^{6}Sig score. NMF: nonnegative matrix factorization; RFE: recursive

feature elimination; PCA: principal component analysis. **B.** m6A Signature score (n=53): Upper panel, distribution of samples in the groups with high and low m^{6}Sig score; middle panel, OS of each sample; lower panel, the expression pattern of 16-gene signature in the two groups. **C-D.** ROC curve shows the specificity and sensitivity of the m6Sig score in our sequencing cohort (n=53) and in TCGA cohort (n=107). **E.** Mutation frequencies of m6A regulators in 537 CLL samples from cBioPortal (https://www.cbioportal.org/study?id=cll_broad_2015).

Figure S2

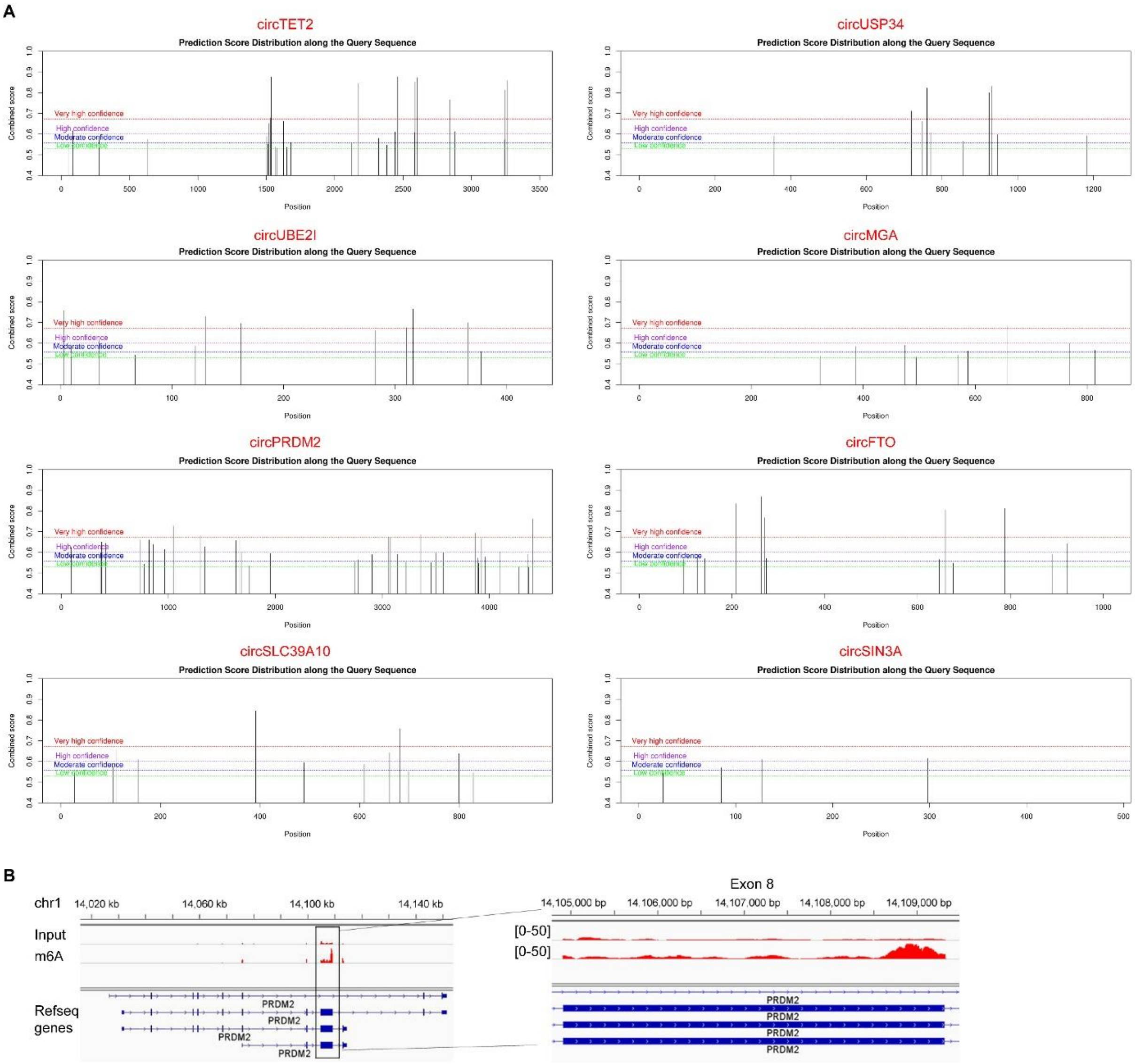

Figure S2. m6A levels of eight circRNAs in our risk model. A. The m6A modification sites and levels of eight circRNAs as predicted by SRAMP

(http://www.cuilab.cn/sramp/). **B.** m6A peak of meRIP-seq on PRDM2 exon 8 as visualized by IGV.

Figure S3

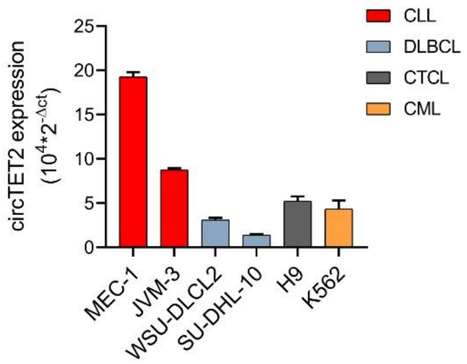

Figure S3. The expression levels of circTET2 in six cell lines from four types of cancers. CLL, chronic lymphocytic leukemia; DLBCL, diffuse large B cell lymphoma; CTCL, cutaneous T-cell lymphoma; AML, acute myeloid leukemia. Error bars represent the means±SD derived from three independent experiments.

Figure S4

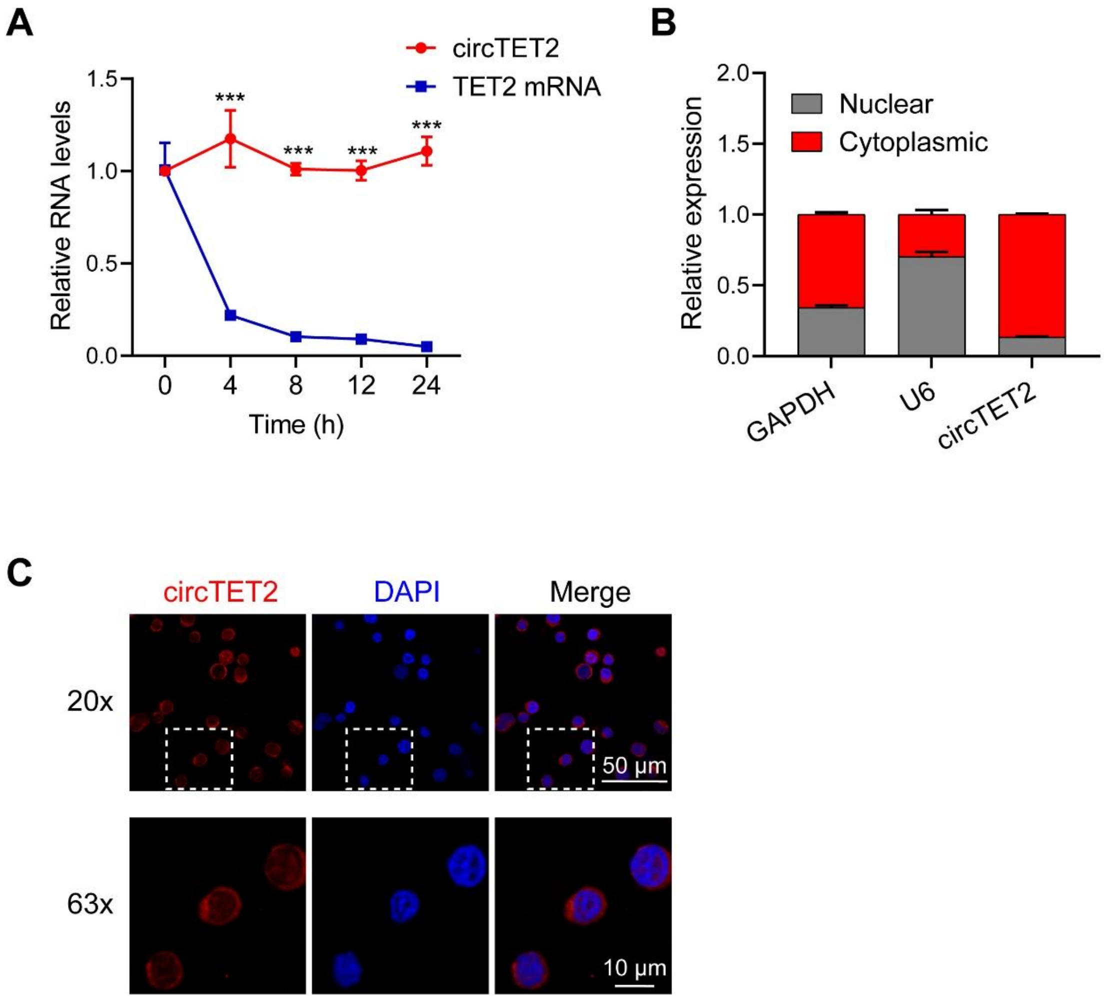

Figure S4. Characterization of circTET2 in JVM-3 cells. A. The abundances of circTET2 and linear TET2 with actinomycin D treatment in JVM-3 cells. **B.** Nucleocytoplasmic separation assays detected the distribution of circTET2 in JVM-3 cells. **C.** FISH assay shows the location of circTET2 in JVM-3 cells. Scale bar, Upper: 50 μm, Lower: 10 μm. Error bars represent the means ± SD derived from three independent experiments. Statistical analyses were performed using a two-tailed Student's t-test, ***$P < 0.001$.

Figure S5

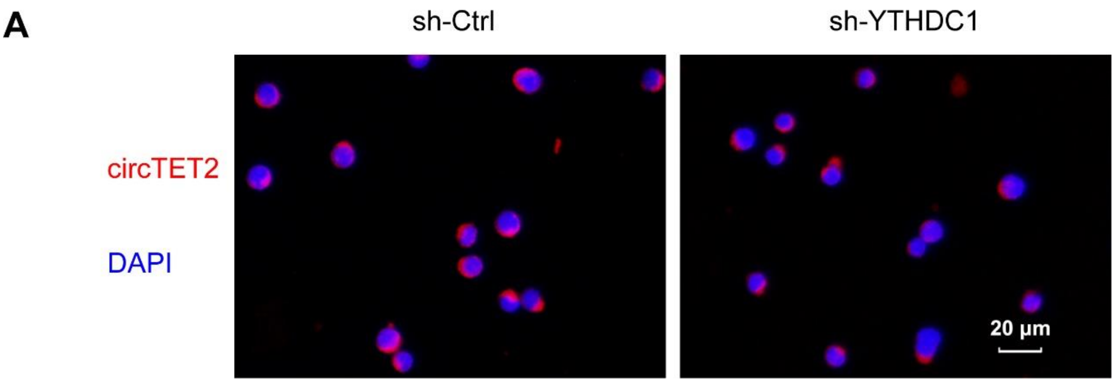

Figure S5. The location of circTET2 after YTHDC1 knockdown in MEC-1 cells (scale bar, 10 μm).

Figure S6

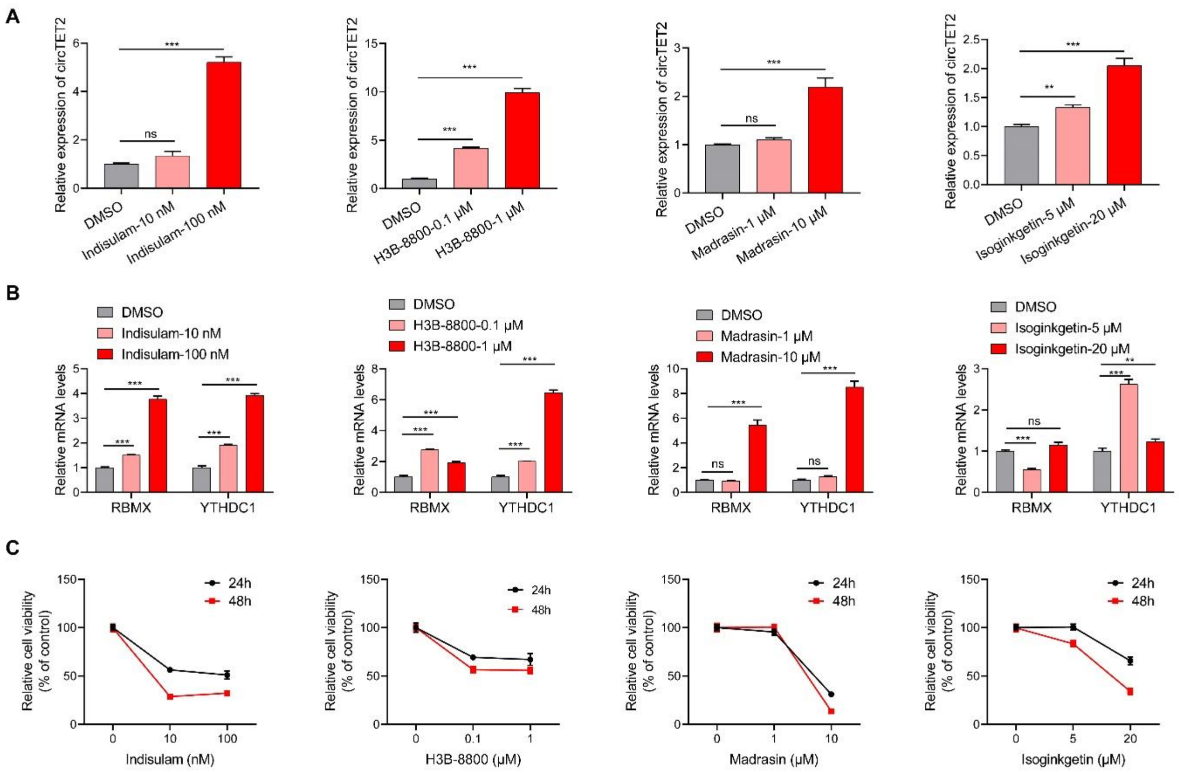

Figure S6. Effects of the modulation of alternative splicing on MEC-1 cells. A. The change in circTET2 levels with inhibitor treatment for 24 h. **B.** The change in RBMX and YTHDC1 with inhibitor treatment for 24 h. **C.** Viability of MEC-1 cells treated with splicing inhibitors as determined by CCK8 assay. Data represent the mean ± SD

from three independent experiments. ns, not significant, * $P < 0.05$, ** $P < 0.01$, *** $P < 0.001$.

Supplementary Tables

Table S1. Proteins pulled down by circTET2 probe.

**Accession**	**-10lgP**	**Coverage (%)**
P04264|K2C1_HUMAN	298.61	56
P13645|K1C10_HUMAN	296.15	56
P13647|K2C5_HUMAN	230.78	29
P68104|EF1A1_HUMAN	226.19	35
P05165|PCCA_HUMAN	212.3	38
P07437|TBB5_HUMAN	211.01	42
P08779|K1C16_HUMAN	202.61	39
P68371|TBB4B_HUMAN	202.48	38
P48668|K2C6C_HUMAN	192.02	25
P68363|TBA1B_HUMAN	189.31	34
Q05639|EF1A2_HUMAN	185.14	22
P08238|HS90B_HUMAN	182.28	21
P60709|ACTB_HUMAN	180.59	36
P00338|LDHA_HUMAN	171.35	35
P78527|PRKDC_HUMAN	168.21	4
P68366|TBA4A_HUMAN	166.82	27
P13639|EF2_HUMAN	166.2	15
P04406|G3P_HUMAN	151.65	33
P06733|ENOA_HUMAN	146.87	24
P14618|KPYM_HUMAN	146.3	21
O15027|SC16A_HUMAN	144.45	5
P07900|HS90A_HUMAN	144.14	11
P13489|RINI_HUMAN	142.49	18

P42704|LPPRC_HUMAN	140.25	5
P12236|ADT3_HUMAN	135.15	20
P27708|PYR1_HUMAN	132.15	5
P60174|TPIS_HUMAN	130.59	31
Q13162|PRDX4_HUMAN	126.87	30
Q00610|CLH1_HUMAN	126.07	7
P07195|LDHB_HUMAN	125.08	16
P62241|RS8_HUMAN	124.14	25
Q7RTS7|K2C74_HUMAN	121.36	9
Q06830|PRDX1_HUMAN	115.92	26
P00558|PGK1_HUMAN	115.5	8
P11142|HSP7C_HUMAN	114.23	16
P39023|RL3_HUMAN	105.34	16
P19338|NUCL_HUMAN	104.55	5
P61313|RL15_HUMAN	99.77	16
Q15365|PCBP1_HUMAN	98.72	18
P14625|ENPL_HUMAN	97.5	4
P11021|BIP_HUMAN	97.35	5
P50914|RL14_HUMAN	97	11
P16403|H12_HUMAN	89.86	18
P25705|ATPA_HUMAN	89.09	8
P26641|EF1G_HUMAN	88.65	9
P49327|FAS_HUMAN	88.62	2
P62753|RS6_HUMAN	88.04	8
P36578|RL4_HUMAN	87.03	5
P07814|SYEP_HUMAN	86.02	2
P62826|RAN_HUMAN	85.6	25
Q15154|PCM1_HUMAN	82.98	1
Q02878|RL6_HUMAN	82.08	8

Q00839|HNRPU_HUMAN	80.28	7
P62987|RL40_HUMAN	79.55	23
Q07020|RL18_HUMAN	78.92	13
P15924|DESP_HUMAN	78.15	1
P14136|GFAP_HUMAN	77.8	5
P46781|RS9_HUMAN	75.68	18
P47929|LEG7_HUMAN	72.9	17
P31146|COR1A_HUMAN	71.9	4
Q86X67|NUD13_HUMAN	71.88	6
P27348|1433T_HUMAN	69.1	12
P27635|RL10_HUMAN	68.95	10
Q02543|RL18A_HUMAN	68.76	13
P10809|CH60_HUMAN	68.46	5
P30740|ILEU_HUMAN	66.8	4
P61978|HNRPK_HUMAN	65.41	6
P06576|ATPB_HUMAN	65.09	3
P06702|S10A9_HUMAN	63.11	25
P62701|RS4X_HUMAN	62.87	7
Q9UQ35|SRRM2_HUMAN	61.23	1
P23396|RS3_HUMAN	60.49	9
P40926|MDHM_HUMAN	58.9	3
P26373|RL13_HUMAN	58.43	8
P05109|S10A8_HUMAN	58.36	24
P13796|PLSL_HUMAN	57.71	4
P99999|CYC_HUMAN	57.45	10
P06276|CHLE_HUMAN	56.98	2
P04075|ALDOA_HUMAN	56.66	8
P40429|RL13A_HUMAN	56.32	5
P83731|RL24_HUMAN	56.19	5

P0DOX6|IGM_HUMAN	53.28	1
P15259|PGAM2_HUMAN	52.95	4
P80297|MT1X_HUMAN	52.55	20
O75445|USH2A_HUMAN	52	0
A2NJV5|KV229_HUMAN	51.62	11
P63104|1433Z_HUMAN	51.4	9
P18124|RL7_HUMAN	51.01	4
A4UGR9|XIRP2_HUMAN	50.97	1
Q12802|AKP13_HUMAN	49.9	0
Q9H1E3|NUCKS_HUMAN	49.79	4
P63244|RACK1_HUMAN	49.54	3
P12273|PIP_HUMAN	49.35	8
P04054|PA21B_HUMAN	48.35	5
P07910|HNRPC_HUMAN	46.52	3
P81605|DCD_HUMAN	45.27	10
P62917|RL8_HUMAN	44.9	4
Q14240|IF4A2_HUMAN	44.59	2
O00571|DDX3X_HUMAN	44.54	3
Q9P2E9|RRBP1_HUMAN	40.03	0
P01040|CYTA_HUMAN	38.46	19
P84098|RL19_HUMAN	37.9	5
P15880|RS2_HUMAN	37.59	4
Q8WWU5|TCP11_HUMAN	37.28	2
P38646|GRP75_HUMAN	37.08	1
P11586|C1TC_HUMAN	37.04	1
Q8TDN2|KCNV2_HUMAN	36.42	1
Q8WVT3|TPC12_HUMAN	36.06	1
Q9C0C4|SEM4C_HUMAN	35.95	1
Q86Y56|DAAF5_HUMAN	35.6	2

Q8N196|SIX5_HUMAN	35.18	2
Q96HU8|DIRA2_HUMAN	34.73	6
A7KAX9|RHG32_HUMAN	33.48	2
P24855|DNAS1_HUMAN	33.07	4
Q7Z6R9|AP2D_HUMAN	33.01	2
O75410|TACC1_HUMAN	32.36	2
P14543|NID1_HUMAN	32.35	1
P30414|NKTR_HUMAN	32.23	1
P08865|RSSA_HUMAN	31.87	6
P21796|VDAC1_HUMAN	31.77	4
P53618|COPB_HUMAN	31.7	1
Q00325|MPCP_HUMAN	31.45	3
A0A2R8Y619|H2BE1_HUMAN	31.36	7
Q9UHM6|OPN4_HUMAN	31.29	1
Q86U44|MTA70_HUMAN	31.23	1
Q13596|SNX1_HUMAN	30.21	2
O15021|MAST4_HUMAN	29.3	0
Q9NSK0|KLC4_HUMAN	29.19	1
Q9P0W8|SPAT7_HUMAN	29.03	1
O60504|VINEX_HUMAN	28.61	2
Q66GS9|CP135_HUMAN	27.61	1
Q7Z6P3|RAB44_HUMAN	27.32	1
P26038|MOES_HUMAN	26.96	1
O00148|DX39A_HUMAN	26.94	2
Q8IWZ3|ANKH1_HUMAN	26.77	0
P02042|HBD_HUMAN	26.52	6
P13010|XRCC5_HUMAN	26.44	1
B0I1T2|MYO1G_HUMAN	26.05	1
Q9NZT1|CALL5_HUMAN	24.66	5

Q96NS5|ASB16_HUMAN	24.38	2
Q13367|AP3B2_HUMAN	24.27	1
Q92621|NU205_HUMAN	24.26	0
P47901|V1BR_HUMAN	24.09	5
Q9C040|TRIM2_HUMAN	23.65	1
Q460N5|PAR14_HUMAN	23.52	0
P42702|LIFR_HUMAN	23.44	1
Q13615|MTMR3_HUMAN	23.25	1
Q96DN5|TBC31_HUMAN	22.78	1
O00329|PK3CD_HUMAN	22.01	1
P01834|IGKC_HUMAN	21.83	16
Q8IYW2|CFA46_HUMAN	21.58	0
Q8IVV2|LOXH1_HUMAN	21.4	0
Q6ZMW2|ZN782_HUMAN	20.98	4
P51795|CLCN5_HUMAN	20.85	4
Q5GH76|XKR4_HUMAN	20.76	3
O95936|EOMES_HUMAN	20.74	3
Q86SF2|GALT7_HUMAN	20.72	1
Q1XH10|SKDA1_HUMAN	20.7	1
Q8IWR1|TRI59_HUMAN	20.02	1

Table S2. Sequences of shRNAs and primers used in this study.

**Name**	**sequences**
sh-RBMX-1	5'- CATCAAGAGGATATAGCGATA-3'
sh-RBMX-2	5'- GACTATCCATCAAGAGGATAT-3'
sh-RBMX-3	5'- GAGGATATAGCGATAGAGATG-3'
sh-YTHDC1-1	5'- GGAAGAAGTGAACTCTGAAGA-3'
sh-YTHDC1-2	5’-GGAAGAGAGCTAGAGGCATAT-3’
sh-YTHDC1-3	5’-GCGTCGACCAGAAGATTATGA-3’

sh-Ctrl	5'- TTCTCCGAACGTGTCACGT-3'
CD36 F	CTTTGGCTTAATGAGACTGGGAC
CD36 R	GCAACAAACATCACCACACCA
circTET2-F	CTTGCAGATGTGTAGAATTC
circTET2-R	ATGGTATCAGGAATGGACTT
CPT1A-F	GATCCTGGACAATACCTCGGAG
CPT1A-R	CTCCACAGCATCAAGAGACTGC
CPT1B-F	TGTATCGCCGTAAACTGGACCG
CPT1B-R	TGTCTGAGAGGTGCTGTAGCAC
GAPDH-F	CTGGGCTACACTGAGCACC
GAPDH-R	AAGTGGTCGTTGAGGGCAATG
HNRNPC-F	TGGGCTGCTCTGTTCATAAGGG
HNRNPC-R	CTCGGTTCACTTTTGGCTCTGC
RBMX-F	AGACGCTAAGGATGCAGCCAGA
RBMX-R	CCACCTCTAAGACCTCTTGGAG
TET2-F	GCTTACCGAGACGCTGAGGAAA
TET2-R	AGAGAAGGAGGCACCACAGGTT
U6-F	CTCGCTTCGGCAGCACA
U6-R	AACGCTTCACGAATTTGCGT
YTHDC1-F	TCAGGAGTTCGCCGAGATGTGT
YTHDC1-R	AGGATGGTGTGGAGGTTGTTCC

Table S3. Small molecular inhibitors and reagents used in this study.

**Reagents**	**Resources**
actinomycin D	MedchemExpress
RNase R	Epicentre Technologies
**Inhibitors**	
CP028	Gift from Yigong Shi
C75	Selleck, S9819

Dactolisib	MedchemExpress, HY-50673
Dorsomorphin	Selleck, S7306
Etomoxir	MedchemExpress, HY-50202
Ezetimibe	Selleck, S1655
FB23-2	MedchemExpress, HY-127103
Firsocostat	Selleck, S8893
H3B-8800	DC Chemicals, 1825302-42-8
Indisulam	Selleck, S9742
Isoginkgetin	Selleck, S5813
Madrasin	Selleck, S0280
Orlistat	MedchemExpress, HY-B0218
Perhexiline maleate	MedchemExpress, HY-B1334A
